# Association of Serum Cardiac Troponin in the Outcome of Acute Myocardial Infarction

**DOI:** 10.7759/cureus.112569

**Published:** 2026-07-13

**Authors:** Ramyashree Reddy, Samaga LN

**Affiliations:** 1 General Medicine, KS Hegde Medical Academy, Nitte University, Mangalore, IND

**Keywords:** acute myocardial infarction, highly sensitive cardiac troponin, hypotension, mortality, reinfarction, wall motion score index

## Abstract

Background

The diagnosis of acute myocardial infarction (AMI) relies on a comprehensive clinical framework that integrates patient presentation, electrocardiographic findings, and cardiac biomarkers, including highly sensitive troponins. While cardiac biomarkers such as highly sensitive troponins have traditionally served as a cornerstone for the diagnosis of acute myocardial infarction, they also hold significant clinical value in risk stratification and prognosis. Highly sensitive troponins may have prognostic value for predicting the course of a patient's hospital stay, including complications and mortality.

Objective

This study aimed to evaluate the association between elevated cardiac troponin levels and clinical outcomes, including hypotension requiring inotropes (indicative of early cardiogenic shock), reinfarction, Wall Motion Score Index (WMSI), and in-hospital mortality, among patients admitted with acute myocardial infarction.

Methods

This prospective observational cohort study was conducted from October 2022 to April 2024 in the department of general medicine at a tertiary care center. A total of 148 patients with acute myocardial infarction were selected using consecutive sampling. Clinical data, including highly sensitive cardiac troponin I (hs-cTnI) and transthoracic echocardiography findings, were collected along with patient demographics, medical histories, and course in the hospital. Blood samples were obtained on admission under aseptic conditions to measure highly sensitive cardiac troponin I. A transthoracic echocardiogram was performed by the same skilled technician to calculate the left ventricular ejection fraction (LVEF) and Wall Motion Score Index (WMSI) using a standard 16-segment model. The course in the hospital was monitored. Statistical analysis was performed using SPSS version 26 (IBM Corp., Armonk, NY), employing descriptive statistics, Spearman correlation coefficients, and the Mann-Whitney test.

Results

The study included 148 patients (66.9% male; mean age: 64.53 years). Median admission hs-cTnI was 1832 ng/L (IQR: 253.2-5887), mean WMSI was 2.04, and mean LVEF was 39.22%. During hospitalization, incidence rates of hypotension, reinfarction, and mortality were 40.5% (n = 60), 8.1% (n = 12), and 18.9% (n = 28), respectively. Admission hs-cTnI positively correlated with WMSI (Spearman's rho = 0.280; 95% CI: 0.124-0.422; p = 0.0006). On ROC analysis, higher values of hs-cTnI were associated with hypotension (AUC = 0.605 (95% CI: 0.508-0.702), p = 0.035; cut-off: 9116 ng/L; sensitivity: 35.0% (95% CI: 23.1%-48.4%), specificity: 93.2% (95% CI: 85.8%-97.4%), PPV: 77.8% (95% CI: 57.7%-91.4%), NPV: 67.8% (95% CI: 58.7%-76.0%)) and reinfarction (AUC = 0.678 (95% CI: 0.511-0.845), p = 0.035; sensitivity: 58.3% (95% CI: 27.7%-84.8%), specificity: 86.8% (95% CI: 80.0%-92.0%), PPV: 28.0% (95% CI: 12.1%-49.4%), NPV: 95.9% (95% CI: 90.8%-98.7%)). Baseline hs-cTnI did not correlate with in-hospital mortality (AUC = 0.513 (95% CI: 0.395-0.631), p = 0.814).

Conclusion

This study supports the potential role of hs-cTnI as an early marker associated with selected in-hospital complications after acute myocardial infarction. This study supports the potential role of highly sensitive cardiac troponin levels as an early marker to assess the risk of myocardial infarction-related complications. Further multicenter, prospective studies and serial measurements of troponin levels are required to validate these findings.

## Introduction

Cardiovascular diseases are the leading cause of mortality worldwide, with acute myocardial infarction (AMI) representing the most severe and life-threatening manifestation of coronary artery disease [1]. In India, the epidemiological shift over the past few decades has resulted in a silent epidemic of cardiovascular disease, characterized by an increasing incidence of AMI among both urban and rural populations [2].

The diagnosis and risk stratification of AMI have evolved significantly with the transition from conventional cardiac troponin assays to highly sensitive cardiac troponin (hs-cTn) assays [3]. Highly sensitive cardiac troponin I (hs-cTnI) enables the detection of myocardial necrosis at much lower concentrations and earlier time points than previous generations of biomarkers [3]. While the diagnostic superiority of hs-cTnI is well established, its prognostic utility, specifically its ability to predict the immediate clinical course and acute hemodynamic or ischemic complications during a patient's hospital stay, remains an area of active investigation [3]. The primary objectives in the management of acute coronary syndrome (ACS) are the rapid restoration of myocardial perfusion, the limitation of infarct size, and the prevention of major adverse cardiovascular events (MACE) [4]. Prior studies have predominantly focused on the biomarker's long-term predictive power for major adverse cardiovascular events (MACE) or mortality in tightly controlled clinical trial datasets. However, there is a paucity of real-world evidence evaluating how a single baseline admission hs-cTnI concentration correlates directly with immediate, acute in-hospital complications such as early hemodynamic collapse requiring inotropic support and detailed regional wall motion scoring systems such as the Wall Motion Score Index (WMSI) at the bedside, particularly in high-acuity tertiary care settings with delayed presentations. This highlights a clear knowledge gap regarding the acute triage utility of the biomarker.

Risk stratification in the acute setting relies on both biochemical markers and imaging. Echocardiography plays an important role in this assessment. While left ventricular ejection fraction (LVEF) is a standard measure of systolic function, it evaluates global volumetric changes and can frequently mask severe, localized myocardial injury due to compensatory hyperkinesis of non-infarcted segments. Conversely, the Wall Motion Score Index (WMSI) employs a detailed segmental analysis that directly quantifies regional contractility abnormalities. This allows WMSI to provide a more granular, anatomically precise assessment of regional dysfunction, serving as a reliable surrogate for infarct size even in patients with relatively preserved global ejection fractions [4]. Understanding the relationship between the magnitude of initial biomarker elevation (hs-cTnI) and echocardiographic parameters (WMSI and LVEF), as well as clinical outcomes such as hypotension, reinfarction, and mortality, is crucial. These specific outcomes were selected to capture a comprehensive, multi-dimensional profile of acute post-MI status. WMSI was utilized as a sensitive structural surrogate to quantify localized regional myocardial necrosis; hypotension requiring inotropic support was monitored as a vital clinical marker of early cardiogenic shock and hemodynamic failure; recurrent in-hospital reinfarction was tracked to evaluate acute ischemic instability; and in-hospital mortality was assessed as the definitive, hard safety endpoint. This study was undertaken to evaluate the association of baseline serum hs-cTnI levels with WMSI, hypotension requiring inotropes (suggestive of early cardiogenic shock), reinfarction, and in-hospital mortality in patients presenting with AMI.

## Materials and methods

Study design and setting

A prospective observational cohort study was conducted at the department of general medicine at a tertiary care center in Mangaluru, South India. The study was carried out over a period of 18 months, from October 2022 to April 2024.

Sample size calculation and sampling technique

The sample size for this study was determined based on the estimated prevalence of acute coronary syndrome. Assuming an expected prevalence (p) of 10.2% based on geographical epidemiological data by Yu et al. [5], a 95% confidence level (Z = 1.96), and a margin of error/precision (d) of 5% (0.05), the initial sample size (n) was calculated using the standard Cochrane formula:

n = (Z² × p × q) / d²

where q = 1 - p (0.898). This calculation yielded a minimum required sample size of 141 patients. To account for potential confounding factors and missing data, the sample size was increased by approximately 5%, resulting in a final enrolled sample size of 148 patients. While this prevalence-based calculation was selected a priori to ensure epidemiological representation of the local AMI cohort, a post hoc power analysis demonstrated that the final sample size of 148 patients achieved 93.4% statistical power (at a two-tailed α of 0.05) to detect the primary correlation (ρ = 0.280) between hs-cTnI and WMSI.

Inclusion and exclusion criteria

The study population comprised 148 patients admitted with a confirmed diagnosis of acute myocardial infarction. Participants were selected using a convenience sampling technique. A diagnosis of myocardial infarction was made based on the Fourth Universal Definition of Myocardial Infarction when at least one of the following signs of myocardial ischemia was present: development of pathological Q waves, new ischemic electrocardiogram (ECG) changes, clinical symptoms, imaging evidence of a new loss of viable myocardium, or new regional wall motion abnormality in a pattern consistent with ischemic etiology, and elevated cardiac troponins [6]. The inclusion criteria mandated the enrolment of adult patients admitted with AMI, encompassing both ST-elevation myocardial infarction (STEMI) and non-ST-segment elevation myocardial infarction (NSTEMI) classifications. Both presentation types were included in a unified acute myocardial infarction cohort to evaluate the prognostic performance of baseline hs-cTnI across the spectrum of acute myocardial necrosis, representing a pragmatic approach to real-world clinical triage in high-acuity settings. Patients with alternative causes of troponin elevation (such as pulmonary embolism, chronic kidney disease, or sepsis) or those who did not consent to participate were excluded. Ethical clearance was obtained from the Institutional Ethics Committee of KS Hegde Medical Academy (KSHEMA) (approval number: EC/NEW/INST/2020/834) before the commencement of the study, and informed consent was acquired from all participants or their legally authorized representatives.

Data collection

On admission, anonymized data were acquired, which included demographic data, clinical history, examination, provisional diagnosis, laboratory measurements, and imaging. Highly sensitive troponin I levels were measured at the time of presentation to the hospital (within 2-3 hours of hospital presentation) by collecting 3 mL blood sample in a green-topped vacutainer under aseptic precautions and were measured using the Beckman Coulter Access hs-cTnI immunoassay, featuring a 99th percentile upper reference limit (URL) of 20 ng/L and a coefficient of variation (CV) of <10%. To ensure consistency and eliminate inter-observer variability, a transthoracic two-dimensional (2D) echocardiogram was performed on all patients within 24 hours of admission by the same skilled echocardiography technician (with over five years of clinical experience), who was strictly blinded to the patients' admission and highly sensitive cardiac troponin I levels to eliminate interpreter bias during the hospital stay. The left ventricular ejection fraction (LVEF) was calculated. The Wall Motion Score Index (WMSI) was derived using the standard 16-segment model of the left ventricle. Each segment was analyzed and scored based on its contractility: 1 = normokinesia, 2 = hypokinesia, 3 = akinesia, and 4 = dyskinesia. The WMSI was calculated by dividing the sum of the scores of all visualized segments by the total number of segments evaluated. The patient's blood pressure was assessed immediately upon arrival and subsequently monitored at six-hour intervals, and their need for inotropes was monitored. Hypotension was defined as systolic blood pressure less than 90 mmHg or mean arterial pressure less than 65 mmHg, and unresponsive to a fluid challenge test suggestive of cardiogenic shock. The patient's hospital stay was monitored for any new-onset chest pain or discomfort. If present, the repeat cardiac troponin sample was analyzed within 2-3 hours of symptoms for reinfarction. A diagnosis of in-hospital reinfarction was established if there was a subsequent rise in troponin levels of more than 20% above a documented baseline value, provided it was accompanied by simultaneous clinical symptoms of ischemia or new ischemic ECG alterations. At the end of the hospital stay, mortality was assessed. In-hospital clinical complications (hypotension, reinfarction, and mortality) were tracked independently as non-mutually exclusive events; however, granular data regarding the precise overlap or co-occurrence of these events within individual patients were not recorded.

Statistical analysis

All collected data were tabulated in Microsoft Excel (Microsoft Corp., Redmond, WA) and statistically analyzed using IBM SPSS Statistics for Windows version 26.0 (IBM Corp., Armonk, NY). Descriptive statistics were used to summarize demographic and baseline clinical variables. Data normality was assessed using the Shapiro-Wilk test. hs-cTnI demonstrated a highly skewed, non-normal distribution; hence, continuous variables involving this biomarker are expressed as medians with interquartile ranges (IQRs). The Spearman correlation coefficient (rho) was utilized to evaluate the correlation between continuous variables, specifically hs-cTnI and WMSI. However, to provide a comprehensive visual representation, data were log-transformed for linear modeling, and both Pearson (on log-transformed data) and Spearman coefficients are reported for robustness. The Mann-Whitney U test was employed to assess the association between hs-cTnI levels and categorical clinical outcomes (hypotension, reinfarction, and mortality). Receiver operating characteristic (ROC) curve analysis was conducted to evaluate the diagnostic and predictive performance of hs-cTnI, calculating and reporting the area under the curve (AUC). The optimal diagnostic and predictive cutoff points for hs-cTnI on the ROC curves were determined by maximizing the Youden Index. To assess the precision of these diagnostic and predictive estimates, 95% confidence intervals (CIs) were calculated for the AUC, sensitivity, specificity, positive predictive value (PPV), and negative predictive value (NPV) using the Wilson score interval method for binomial proportions. Additionally, 95% CIs were calculated for the Spearman correlation coefficient using Fisher's Z-transformation and for the overall mortality rate using binomial proportion intervals. A p-value of <0.05 was considered statistically significant.

## Results

Demographics

The study included a total of 148 patients diagnosed with acute myocardial infarction. The demographic profile demonstrated a male predominance, with 66.9% male patients (n = 99) and 33.1% female patients (n = 49). The mean age of the study cohort was 64.53 years.

Baseline clinical and investigational markers revealed a median admission hs-cTnI level of 1832 ng/L (IQR: 253.2-5887 ng/L). Echocardiographic evaluation demonstrated a mean LVEF of 39.22% ± 13.68% and a mean WMSI of 2.04 ± 0.57 (Table 1).

**Table 1:** Baseline patient characteristics

Characteristic	Reference range	Mean value	Median	Standard deviation	Interquartile range
Age (years)	NA	64.5	64.5	11.84	57, 73
Ejection fraction (%)	55%-70%	39.22	40	13.68	30, 50
Wall Motion Score Index	1.0 (normokinesia)	2.04	2.04	0.57	1.65, 2.42
Cardiac troponin I (ng/L)	0-20 (based on the assay-specific 99th percentile upper reference limit)	6121.87	1832	11338.19	253.2, 5887

Clinical outcomes

A significant proportion of the cohort experienced in-hospital clinical complications. Hypotension requiring the initiation of inotropic support (indicative of early cardiogenic shock) developed in 40.5% of the patients (n = 60). Reinfarction during the hospital stay occurred in 8.1% of the patients (n = 12). The overall in-hospital mortality rate for this cohort was 18.9% (n = 28) (Table 2).

**Table 2:** Clinical outcomes

Outcome	Frequency (number)	Percentage (%)
Hypotension	60	40.5
Reinfarction	12	8.1
In-hospital mortality	28	18.9

Association of troponin with structural and clinical outcomes

A detailed statistical analysis was conducted to evaluate the association between baseline hs-cTnI levels and the predefined clinical and echocardiographic endpoints.

hs-cTnI and WMSI

**Figure 1:**
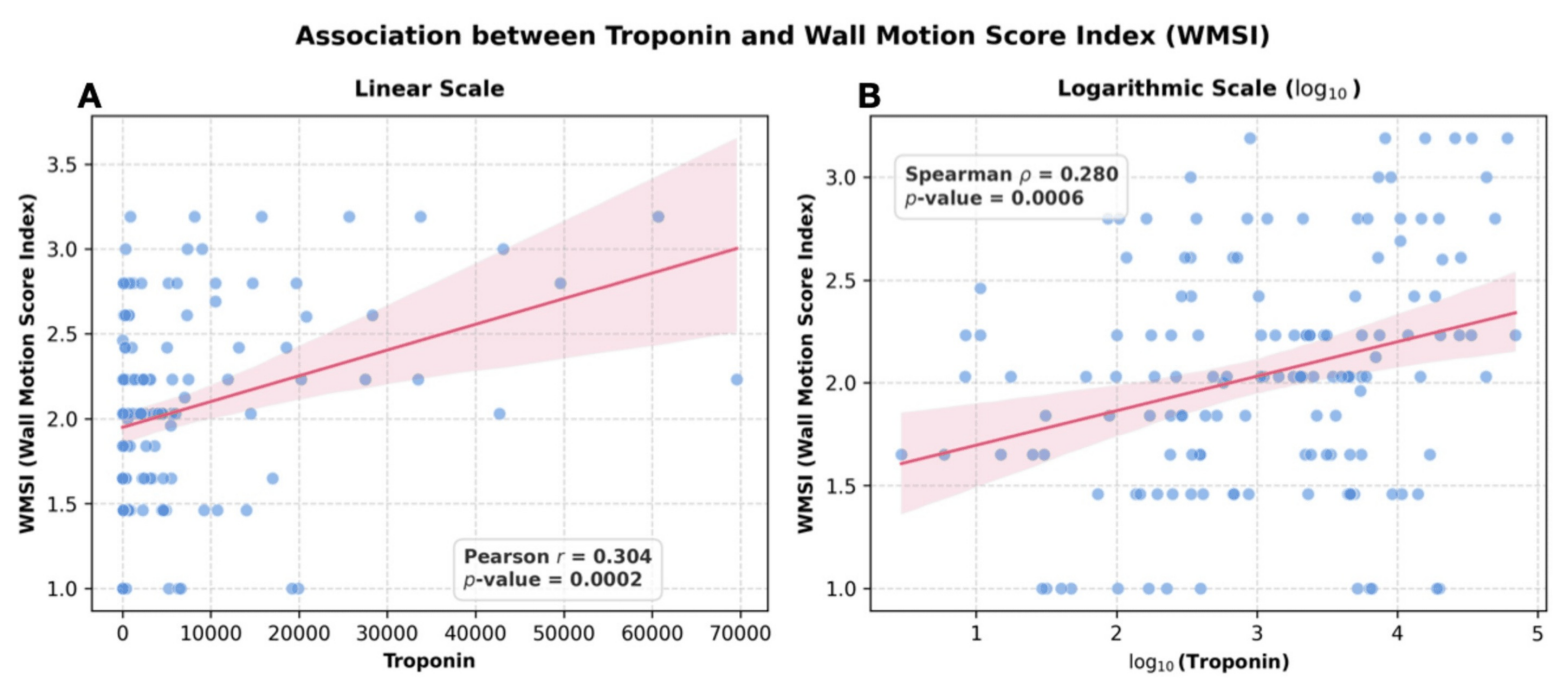
Dual-panel scatterplot illustrating the correlation between baseline highly sensitive cardiac troponin I and WMSI (A) Linear scale representation demonstrating a positive Pearson correlation (r = 0.304, p = 0.0002). (B) Logarithmic scale representation demonstrating a positive Spearman rank correlation (ρ = 0.280, p = 0.0006). WMSI: Wall Motion Score Index

There was a statistically significant positive correlation between admission hs-cTnI levels and WMSI (Spearman's rho = 0.280; 95% CI: 0.124-0.422; p = 0.0006) (Figure 1). This indicates a modest but statistically significant positive correlation between baseline troponin levels and the extent of regional wall motion abnormalities.

hs-cTnI and Hypotension

**Figure 2:**
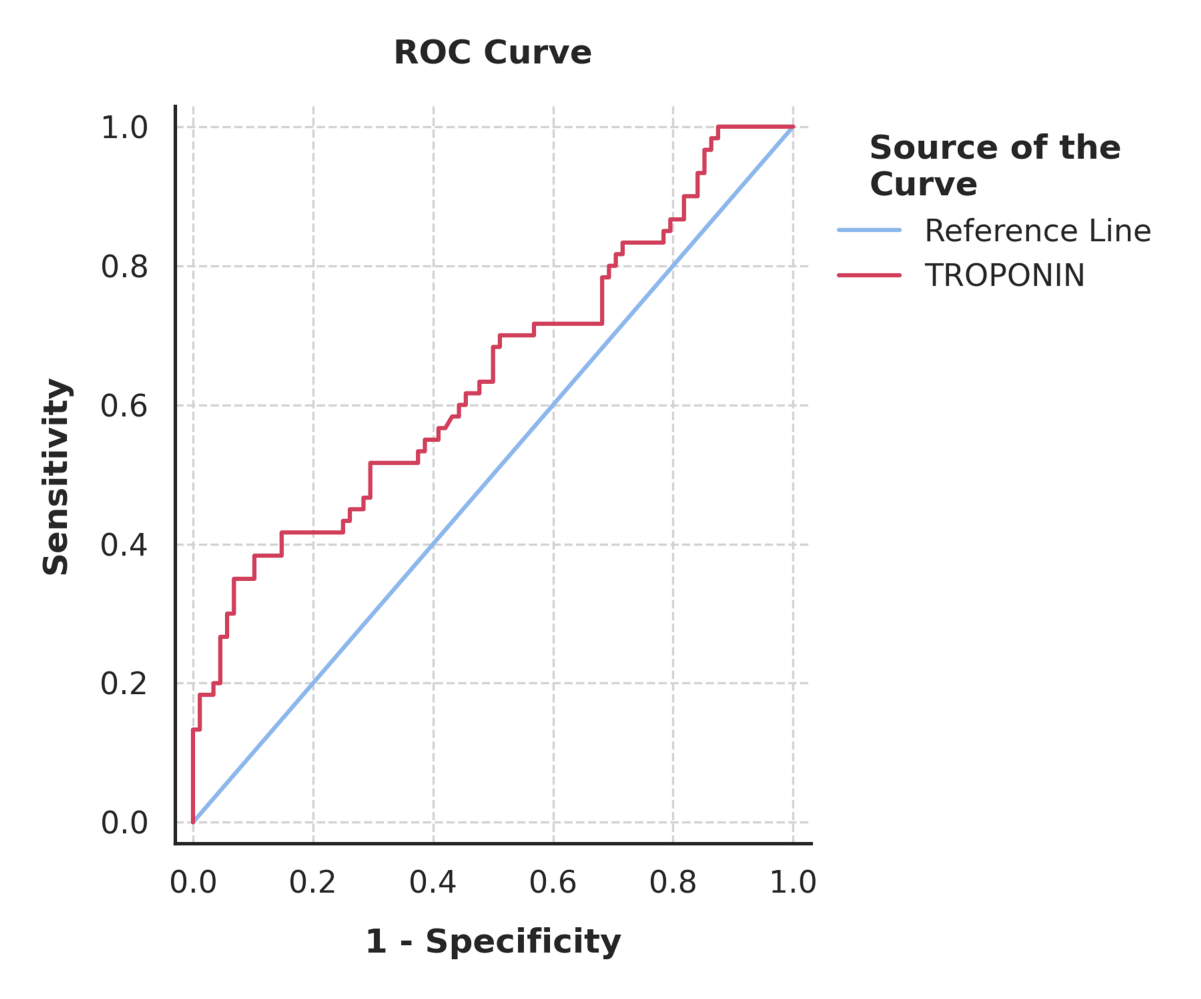
ROC curve demonstrating the capability of highly sensitive cardiac troponin I for predicting in-hospital hypotension among patients with acute myocardial infarction AUC = 0.605, 95% CI: 0.508-0.702, p = 0.035 The optimal exploratory cutoff point of 9116 ng/L was determined by maximizing the Youden Index. ROC: receiver operating characteristic, AUC: area under the curve, CI: confidence interval

A significant positive association was found between baseline hs-cTnI levels and the development of hypotension requiring inotropes (p = 0.035). ROC curve analysis demonstrated an area under the curve (AUC) of 0.605 (95% CI: 0.508-0.702, p = 0.035), identifying a Youden-derived threshold of 9116 ng/L for the development of this complication in this cohort (Figure 2). At this threshold, hs-cTnI demonstrated a sensitivity of 35.0% (95% CI: 23.1%-48.4%), a specificity of 93.2% (95% CI: 85.8%-97.4%), a positive predictive value (PPV) of 77.8% (95% CI: 57.7%-91.4%), and a negative predictive value (NPV) of 67.8% (95% CI: 58.7%-76.0%). Consequently, in this cohort, an hs-cTnI level above 9116 ng/L was associated with a positive predictive value (PPV) of 77.8% for hypotension requiring inotropes (indicative of early cardiogenic shock).

hs-cTnI and Reinfarction

**Figure 3:**
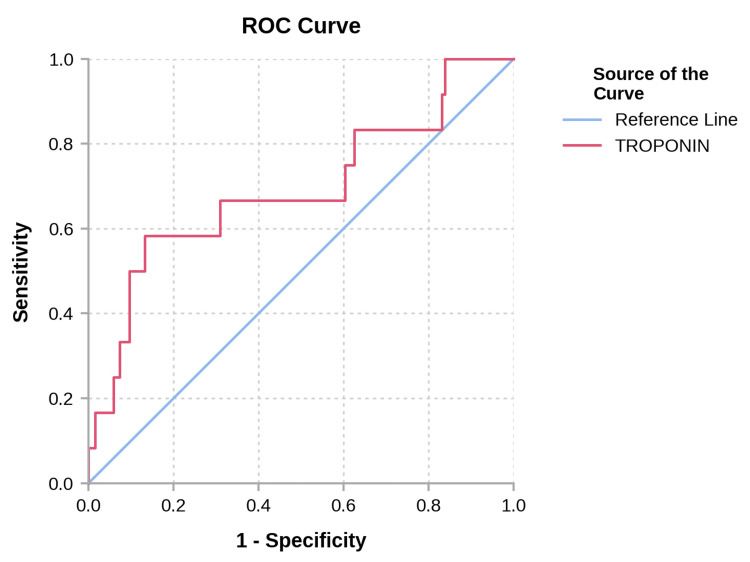
ROC curve demonstrating the capability of admission highly sensitive cardiac troponin I for predicting in-hospital reinfarction among patients with acute myocardial infarction AUC = 0.678, 95% CI: 0.511-0.845, p = 0.035 The optimal exploratory cutoff point of 10,505 ng/L was determined by maximizing the Youden Index. ROC: receiver operating characteristic, AUC: area under the curve, CI: confidence interval

Baseline hs-cTnI was also a statistically significant predictor of in-hospital reinfarction, demonstrating an AUC of 0.678 (95% CI: 0.511-0.845, p = 0.035). Statistical analysis yielded a sensitivity of 58.3% (95% CI: 27.7%-84.8%), a specificity of 86.8% (95% CI: 80.0%-92.0%), a PPV of 28.0% (95% CI: 12.1%-49.4%), and an NPV of 95.9% (95% CI: 90.8%-98.7%) (Figure 3). A troponin cutoff value of 10,505 ng/L proved effective for exclusion of reinfarction in this cohort, demonstrating a negative predictive value of 95.9%. An admission hs-cTnI level above the calculated threshold of 10,505 ng/L was associated with a positive predictive value (PPV) of 28% for a subsequent in-hospital reinfarction in this cohort.

hs-cTnI and Mortality

**Figure 4:**
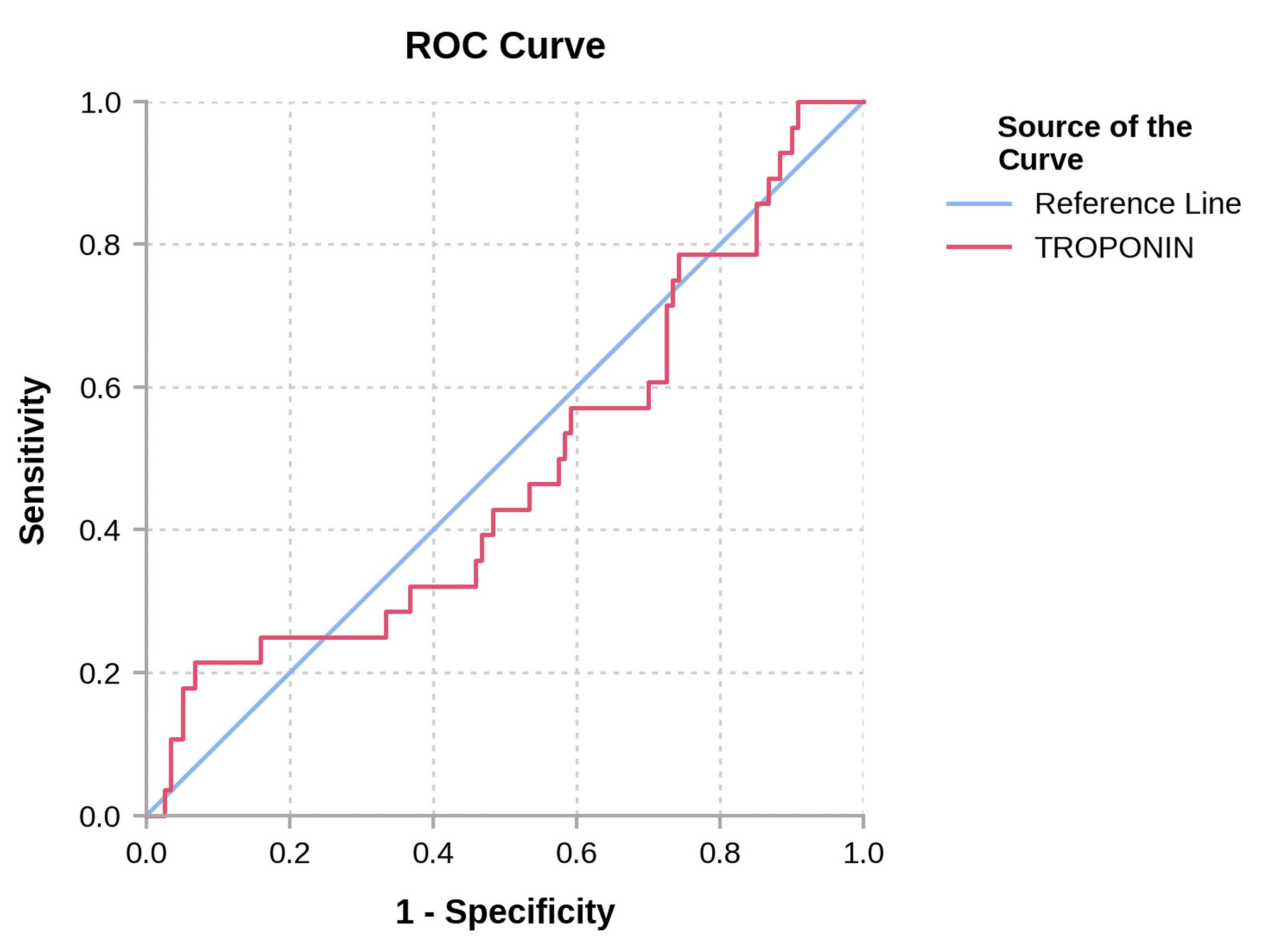
ROC curve demonstrating the diagnostic capability of admission highly sensitive cardiac troponin I for predicting in-hospital mortality among patients with acute myocardial infarction AUC = 0.513, 95% CI: 0.395-0.631, p = 0.814 ROC: receiver operating characteristic, AUC: area under the curve, CI: confidence interval

In contrast to the mechanical and perfusion complications, there was no statistically significant correlation between the baseline hs-cTnI level and in-hospital mortality in this cohort, demonstrating an AUC of 0.513 (95% CI: 0.395-0.631, p = 0.814) (Figure 4). Baseline troponin was not found to be a statistically significant predictor of in-hospital mortality.

## Discussion

The present study evaluated the prognostic capacity of baseline hs-cTnI in predicting immediate in-hospital outcomes among 148 patients with AMI. Our findings demonstrate that while hs-cTnI is associated with indicators of infarct size and hemodynamic instability, it does not independently predict acute in-hospital mortality in this cohort.

The positive correlation between hs-cTnI and WMSI (rho = 0.280; 95% CI: 0.124-0.422; p = 0.0006) suggests that initial troponin release kinetics trend with the magnitude of regional myocardial injury, although the relationship is modest. The mean WMSI in our cohort was notably high at 2.04 (95% CI: 1.95-2.13). In comparison, a study by Jurado-Román et al. [7] reported a lower mean WMSI of 1.46, wherein a WMSI > 1.8 was found to significantly predict both death and hospital readmission. The higher mean WMSI in our study aligns with the substantial rate of in-hospital complications observed, indicating a cohort with a severe baseline burden of myocardial injury. Similarly, the mean LVEF in our study was 39.22%, which is lower than the 52.2% reported by Jurado-Román et al. [7], although slightly higher than the 33.9% observed in a cohort by Tsutamoto et al. [8].

Our data revealed that hs-cTnI is a significant predictor of mechanical and perfusion complications, specifically hypotension requiring inotropes (p = 0.035) and reinfarction (p = 0.035). The calculated exploratory cutoff of 9116 ng/L for hypotension provides a cohort-specific benchmark that may be clinically relevant. With a specificity of 93.2% (95% CI: 85.8%-97.4%) and a PPV of 77.8% (95% CI: 57.7%-91.4%), an admission hs-cTnI value exceeding this threshold may serve as an indicator for clinicians to consider closer hemodynamic monitoring and early risk stratification in an intensive care setting. The high NPV (95.9%) for reinfarction also suggests that lower troponin levels are highly reassuring against the short-term recurrence of ischemic events.

Interestingly, our study found no statistically significant correlation between baseline hs-cTnI and in-hospital mortality (p = 0.814). This finding contrasts with several large-scale studies in the literature. For instance, Antman et al. [9] demonstrated the multicentric efficacy of cTnI in predicting mortality across 1404 patients with AMI. The lack of a statistically significant correlation between baseline hs-cTnI and in-hospital mortality in our study cohort warrants careful consideration, particularly as it contrasts with established literature. This discrepancy may stem from the fact that acute mortality in a contemporary tertiary setting is dictated by multiple parameters, such as immediate mechanical complications, the success and timing of revascularization strategies, and the management of fatal arrhythmias. A single admission biomarker may reflect the initial mass of myocardial necrosis, but cannot dynamically account for subsequent therapeutic successes or multi-organ failure cascades during the acute stay.

A notable finding in our study is the high rate of in-hospital complications, including a 40.5% incidence of hypotension and an 18.9% mortality rate. In such high-acuity, undifferentiated settings, the ability of a single admission biomarker to serve as an indicator of infarct size and hemodynamic instability becomes exceptionally valuable for early ICU triage.

The findings of this study should be interpreted in the context of certain limitations. First, the use of a consecutive sampling strategy at a single center introduces potential sampling and selection biases, which restrict the representativeness of our study population and the external generalizability of the results to broader, more diverse demographic profiles. Consequently, these findings from a single-center tertiary care framework with a limited sample size may not fully generalize to community hospital cohorts or alternative healthcare systems utilizing different acute reperfusion strategies. Additionally, while all patients met the clinical criteria for ischemia upon enrollment, we did not granularly categorize or stratify the baseline clinical presentation types (such as textbook typical angina versus atypical or silent symptoms), which may independently influence clinician triage tracking and downstream complications.

Second, due to the nature of data collection, we lacked granular data regarding the classification of myocardial infarction (STEMI versus NSTEMI), baseline comorbidities, and the specific revascularization strategies employed (e.g., primary percutaneous coronary intervention (PCI) versus thrombolysis versus medical management). Reperfusion strategies profoundly influence troponin washout kinetics, infarct size, and survival, and the inability to adjust for these interventions in a multivariable regression model means that baseline hs-cTnI can only be viewed as an unadjusted, associative risk marker rather than an independent predictor of mortality. While major confounders such as chronic kidney disease were excluded at baseline, other causes of non-ischemic troponin elevation, such as subclinical rhabdomyolysis or active autoimmune flares, were not systematically screened, which remains a minor limitation.

Additionally, the study was confined to a short follow-up period that captured only the immediate in-hospital clinical course and acute complications, precluding the evaluation of long-term cardiovascular outcomes, readmission rates, and overall prognosis. We also acknowledge that our tracking of in-hospital mortality may be statistically underpowered due to the sample size constraint and the distribution of survival events. Consequently, the lack of a statistically significant correlation between baseline hs-cTnI and acute mortality must be interpreted cautiously, as it may represent a type II statistical error resulting from inadequate statistical power rather than a true clinical absence of association. Furthermore, clinical outcomes were monitored as independent endpoints, and the dataset lacks the granularity to determine the exact number of patients who experienced overlapping complications (e.g., patients presenting with both hypotension and subsequent reinfarction). Finally, our analysis was restricted to a single baseline measurement of highly sensitive cardiac troponin I obtained upon admission prior to any therapeutic intervention.

## Conclusions

This study highlights the role of baseline highly sensitive cardiac troponin I (hs-cTnI) as a potential early prognostic tool in the management of acute myocardial infarction. Admission hs-cTnI levels significantly correlate with the Wall Motion Score Index, trending with echocardiographic indicators of regional myocardial injury. Furthermore, our findings underscore the potential utility of admission hs-cTnI in predicting clinical course trends, highlighting its role in future risk stratification models once multicenter validation is achieved. These findings underscore the utility of hs-cTnI in anticipating the clinical course, allowing for timely triage and aggressive monitoring. However, as baseline hs-cTnI did not independently predict acute in-hospital mortality, clinicians must rely on a holistic, multi-parametric approach for comprehensive mortality risk stratification in the acute setting. Further large-scale, multicenter prospective studies are warranted to validate these exploratory thresholds. Future research should focus on evaluating serial hs-cTnI measurement kinetics, investigating multi-marker risk algorithms that combine biochemical and imaging data, and assessing long-term post-discharge clinical outcomes beyond the immediate in-hospital phase.

## References

[1] Roth GA, Mensah GA, Johnson CO (2020). Global burden of cardiovascular diseases and risk factors, 1990-2019: update from the GBD 2019 study. J Am Coll Cardiol.

[2] Rao BH (2014). Global burden of sudden cardiac death and insights from India. Indian Heart J.

[3] Reichlin T, Twerenbold R, Wildi K (2015). Prospective validation of a 1-hour algorithm to rule-out and rule-in acute myocardial infarction using a high-sensitivity cardiac troponin T assay. CMAJ.

[4] Modak V, Satish V, Maliha M, Kumar SS, Christia P (2025). The role of imaging modalities in estimating myocardial viability: a narrative review. J Clin Med.

[5] Yu B, Akushevich I, Yashkin AP, Kravchenko J (2021). Epidemiology of geographic disparities of myocardial infarction among older adults in the United States: analysis of 2000-2017 Medicare data. Front Cardiovasc Med.

[6] Thygesen K, Alpert JS, Jaffe AS, Chaitman BR, Bax JJ, Morrow DA, White HD (2018). Fourth universal definition of myocardial infarction (2018). J Am Coll Cardiol.

[7] Jurado-Román A, Agudo-Quílez P, Rubio-Alonso B (2019). Superiority of wall motion score index over left ventricle ejection fraction in predicting cardiovascular events after an acute myocardial infarction. Eur Heart J Acute Cardiovasc Care.

[8] Tsutamoto T, Kawahara C, Nishiyama K, Yamaji M, Fujii M, Yamamoto T, Horie M (2010). Prognostic role of highly sensitive cardiac troponin I in patients with systolic heart failure. Am Heart J.

[9] Antman EM, Tanasijevic MJ, Thompson B (1996). Cardiac-specific troponin I levels to predict the risk of mortality in patients with acute coronary syndromes. N Engl J Med.

